# Facile one-pot synthesis of polytypic CuGaS_2_ nanoplates

**DOI:** 10.1186/1556-276X-8-524

**Published:** 2013-12-13

**Authors:** Zhongping Liu, Qiaoyan Hao, Rui Tang, Linlin Wang, Kaibin Tang

**Affiliations:** 1 Division of Nanomaterials and Chemistry, Hefei National Laboratory for Physical Sciences at the Microscale; Department of Chemistry, University of Science and Technology of China, Hefei 230026, People’s Republic of China; 2 Shanghai Institute of Applied Physics, Chinese Academy of Sciences, 2019 Jia Luo Road, Jiading District, Shanghai 201800, People’s Republic of China

**Keywords:** CuGaS_2_, Polytypism, Nanoplates, Thermolysis

## Abstract

CuGaS_2_ (CGS) nanoplates were successfully synthesized by one-pot thermolysis of a mixture solution of CuCl, GaCl_3_, and 1-dodecanethiol in noncoordinating solvent 1-octadecene. Their morphology, crystalline phase, and composition were characterized by scanning electron microscopy (SEM), transmission electron microscopy (TEM), high-resolution transmission electron microscopy (HRTEM), powder X-ray diffraction (XRD), and X-ray photoelectron spectroscopy (XPS), respectively. Crystalline structure analysis showed that the as-prepared CGS nanoplates were polytypic, in which the wurtzite phase was interfaced with zincblende domains. The growth process of CGS nanoplates was investigated. It was found that copper sulfide nanoplates were firstly formed and then the as-formed copper sulfide nanoplates gradually transformed to CGS nanoplates with proceeding of the reaction. The optical absorption of the as-synthesized CGS nanoplates was also measured and the direct optical bandgap was determined to be 2.24 eV.

## Background

I-III-VI_2_ semiconductor nanocrystals have received much research interest in recent years because they have low toxicity, high absorption coefficient, narrow bandgap, and tunable emission wavelength in the red to near-infrared region and have shown great potential in many fields such as low-cost solar cells, bio-imaging, light-emitting diodes, and visible-light photocatalyst [1-6]. These compounds have two different metal ions, complex structures, and flexible compositions, so it is a formidable challenge to synthesize their nanomaterials in a controlled manner [7-11].

As a member of the I-III-VI_2_ compounds, CuGaS_2_ (CGS) has a direct bandgap of approximately 2.49 eV for the bulk, and can be applied in green-light emission as well as in visible-light-induced photocatalysis [12,13]. Generally, CGS crystallizes in tetragonal chalcopyrite phase at room temperature, and corresponding nanocrystals were previously synthesized by hydrothermal and solvothermal methods [14-16]. However, the products obtained using these methods are mostly in the form of large crystallites with a board size distribution. Recently, CGS nanocrystals with well-defined sizes and shapes, including quantum dots, tadpole-like nanocrystals, nanorods, and nanoplates, were prepared by several research groups [17-21]. For instance, Tung et al. synthesized chalcopyrite CGS nanorods by irradiating the precursor solution with intense X-rays [17]. In particular, several research groups have synthesized CGS nanocrystals with metastable wurtzite structure which is a cation-disordered phase [18-21]. Wang et al. reported tadpole-like CGS nanocrystals with wurtzite phase by a hot-injection approach [18]. Xiao et al. prepared wurtzite CGS nanorods by the reaction of copper(I) acetate, gallium(III) acetylacetonate, and 1-dodecanethiol (DT) in the solvent 1-octadecene at elevated temperature [19]. However, two-dimensional CGS nanocrystals such as nanoplates are less reported up to now, despite the fact that Kluge et al. obtained CGS nanoplates by bulk thermolysis of complex single-source precursors [21].

In this work, we present a facile one-pot method to synthesize CGS nanoplates, wherein the mixed solution of CuCl, GaCl_3_, and 1-dodecanethiol was thermally decomposed in non-coordinating solvent 1-octadecene at elevated temperature. The crystal phase of the as-prepared CGS nanoplates was revealed to be wurtzite-zincblende polytypism. Their growth process and optical absorption were also investigated.

## Methods

### Materials

CuCl, DT, toluene, and anhydrous ethanol were of analytical grade and purchased from Sinopharm Chemical Reagent Co., Ltd (Shanghai, China); GaCl_3_ (99.999%) was purchased from Alfa Aesar (Wardhill, MA, USA); 1-octadecene (ODE, 90%) was purchased from Aldrich (St. Louis, MO, USA). All the reagents were used as received without any further purification.

### Synthesis of CuGaS_2_ nanoplates

In a typical synthesis, 0.25 mmol CuCl, 0.25 mmol GaCl_3_, 0.5 mL DT, and 5 mL ODE were loaded into a 50-mL three-neck flask in a glovebox. The flask was then attached to a Schlenk line. Prior to heating, the mixture system was cycled between vacuum and nitrogen three times, heated to 90°C and then was vacuumed for 10 min. The flask was then filled with nitrogen and heated to 270°C at a rate of 12°C · min^-1^ with magnetic stirring. After the reaction was allowed to proceed for 40 min, the reaction flask was naturally cooled to room temperature. The resulting CuGaS_2_ nanocrystals were collected by centrifugation and were washed thoroughly with toluene and ethanol. Finally, the purified nanocrystals were dried under vacuum for characterization.

### Characterization

The samples were characterized by powder X-ray diffraction (XRD) on a Philips X'pert X-ray diffractometer (Amsterdam, The Netherlands) equipped with Cu Kα radiation (*λ* =1.5418 Å). Transmission electron microscope (TEM) images were taken with a Hitachi H-7650 microscope at an acceleration voltage of 100 kV. High-resolution transmission electron microscope (HRTEM) images were performed on a JEOL-2010 microscope (Akishima-shi, Japan). The scanning electron microscopy (SEM) images were taken using a Zeiss Supra 40 field emission scanning electron microscope (Oberkochen, Germany) operated at 5 kV. X-ray photoelectron spectra (XPS) were recorded on an ESCALab MKII X-ray photoelectron spectrometer (VG Scienta, Newburyport, MA, USA). The UV–vis absorption spectra were recorded on a Solid Spec-3700 spectrophotometer.

## Results and discussion

**Figure 1:**
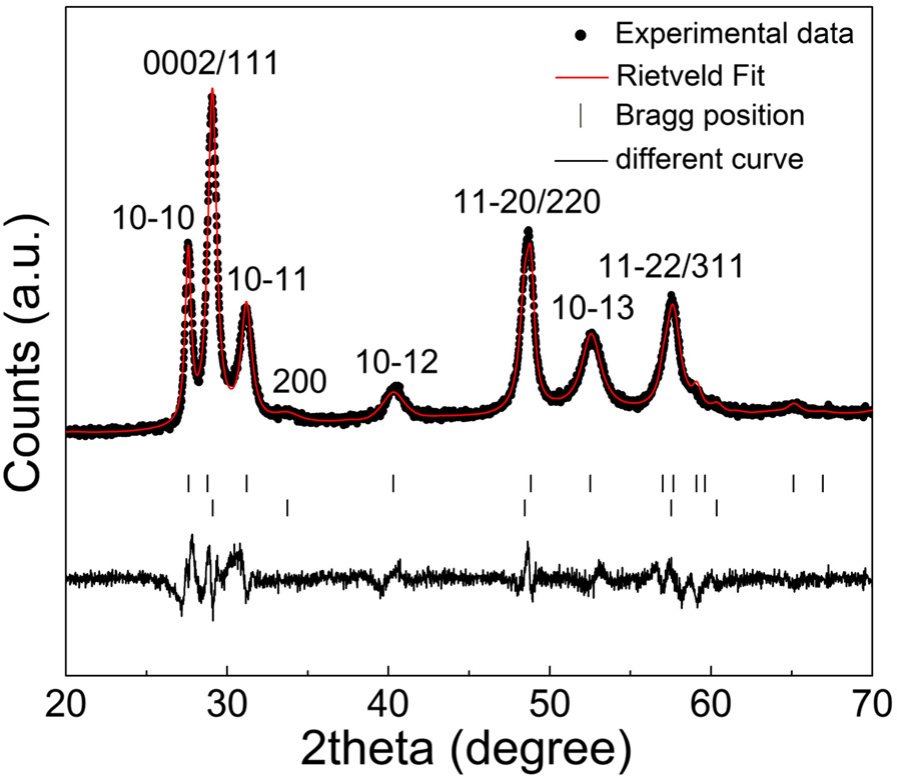
**Powder XRD pattern of as-synthesized product.** The experimental data (dots), a Rietveld fit (red line, Rwp 3.57%, Rp 2.70%), reflection positions of wurtzite (top row) and zincblende (bottom row) CuGaS_2_, and the different curves are displayed.

Figure 1 shows the powder XRD pattern of the as-synthesized product. Generally, CuGaS_2_ (CGS) crystallizes in thermodynamically stable tetragonal chalcopyrite structure, in which Cu and Ga ions are ordered in the cation sublattice sites (Additional file 1: Figure S1a). Meanwhile, two cation-disordered structures, i.e. cubic zincblende modification (Additional file 1: Figure S1b) and hexagonal wurtzite phase (Additional file 1: Figure S1c), can be constructed for CGS [21]. The present XRD pattern was characteristic of a hexagonal wurtzite structure. In addition, a weak reflection peak at 2*θ* = 33.7° was found in the present XRD pattern, which was indexed to (200) of cubic zincblende CGS. Thus, the obtained product also contains cubic zincblende CGS. No characteristic peaks of other impurities such as copper or indium sulfides were observed, which indicates that the as-synthesized product is composed of pure ternary CGS. To determine the lattice parameters and proportions of wurtzite and zincblende structures in the as-synthesized product, the present XRD pattern was well fitted by using Rietveld refinement analysis performed with MAUD program [22]. It is determined that the product consists of approximately 60% hexagonal wurtzite CGS (*P*6_3_*mc*, *a* = 3.727(5) Å, *c* = 6.197(6) Å) and 40% cubic zincblende CGS (*F*-43 *m*, *a* = 5.309(0) Å).

**Figure 2:**
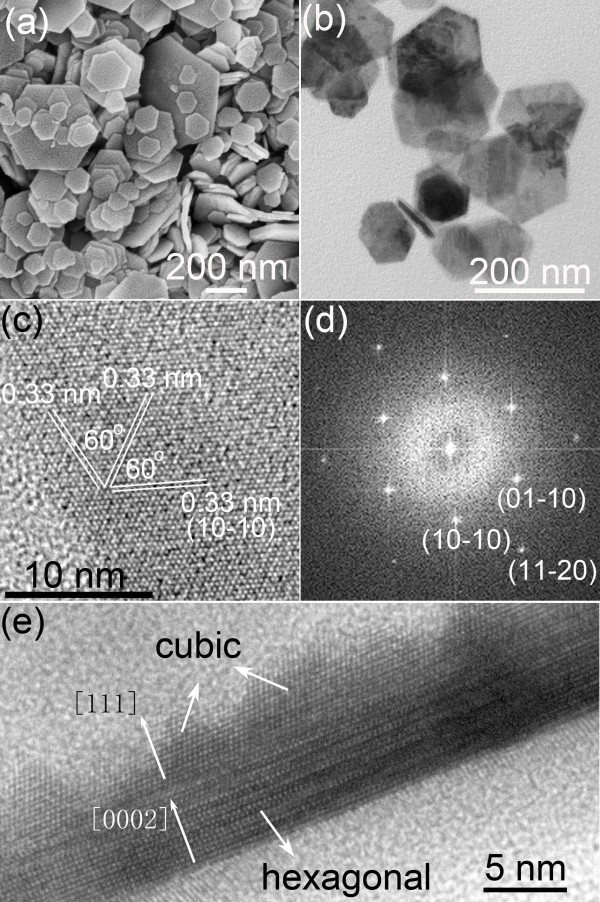
**SEM (a), TEM (b), and HRTEM (c,e) images of as-synthesized product and FFT pattern (d) of (c).** In particular, the HRTEM image **(c)** was taken from the face of nanoplates while the HRTEM image **(e)** was taken from the sides of nanoplates.

The morphologies and size of the as-synthesized product were examined by SEM and TEM. The SEM and TEM images (Figure 2a,b) show that the as-synthesized product consists of hexagonal nanoplates. These nanoplates have a diameter of 70 to 350 nm and a thickness of *ca.* 20 nm. As shown in Figure 2c, the HRTEM image taken from the face of nanoplates exhibits clear lattice fringes with spacings of 0.33 nm, assigning to (10–10) planes of wurtzite CGS. The corresponding FFT pattern (Figure 2d) displays the bright spots with sixfold symmetry, consistent with the hexagonal wurtzite structure of CGS. Furthermore, HRTEM image was also taken from the sides of nanoplates, as shown in Figure 2e. The AB-stacking of the layers in the hexagonal domains and the ABC-stacking in the cubic domains are clearly distinguishable in the HRTEM image shown in Figure 2e, which suggests the coexistence of wurtzite and zincblende structures within each nanoplate. Therefore, the crystal phase of the as-synthesized nanoplates is wurtzite-zincblende polytypism, wherein the hexagonal wurtzite domains are interfaced with the cubic zincblende domains across (0002)_WZ_/(111)_ZB_ stacking faults. This crystal structure of CGS nanoplates is similar to that of our previously synthesized CuInS_2_ nanoplates [23].

**Figure 3:**
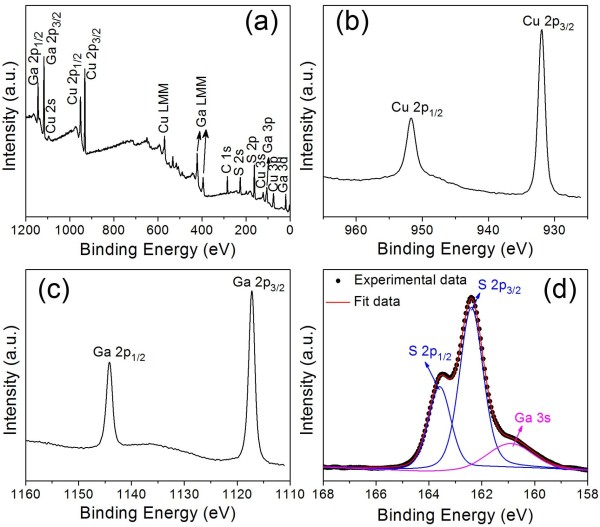
**XPS of as-synthesized nanoplates: (a) a survey spectrum, (b) Cu 2** ***p*, (c) Ga 2** ***p*, and (d) S 2** ***p*.**

The valence states and composition of the as-synthesized nanoplates were studied by XPS, as shown in Figure 3. The full-scan spectra (Figure 3a) show the presence of the Cu 2*p*, Ga 2*p* and S 2*p* peaks, confirming the presence of these elements in as-synthesized nanoplates. The Cu 2*p*, Ga 2*p* and S 2*p* core levels were also examined, respectively. The peaks observed at 931.9 and 951.7 eV, with a peak splitting of 19.8 eV, are indicative of monovalent Cu [23]. The two peaks centered at 1,117 and 1,144 eV, with a peak separation of 27 eV, are attributed to trivalent Ga [20]. The two peaks of S 2*p* were located at 162.4 and 163.6 eV, with a peak splitting of 1.2 eV, which are consistent with the literature values in metal sulfides [24]. Through quantification of peaks, the molar ratio of Cu/Ga/S of 1.22:1:1.93 is given, indicating that the as-synthesized nanoplates are Cu-rich with respect to the stoichiometric CGS.

**Figure 4:**
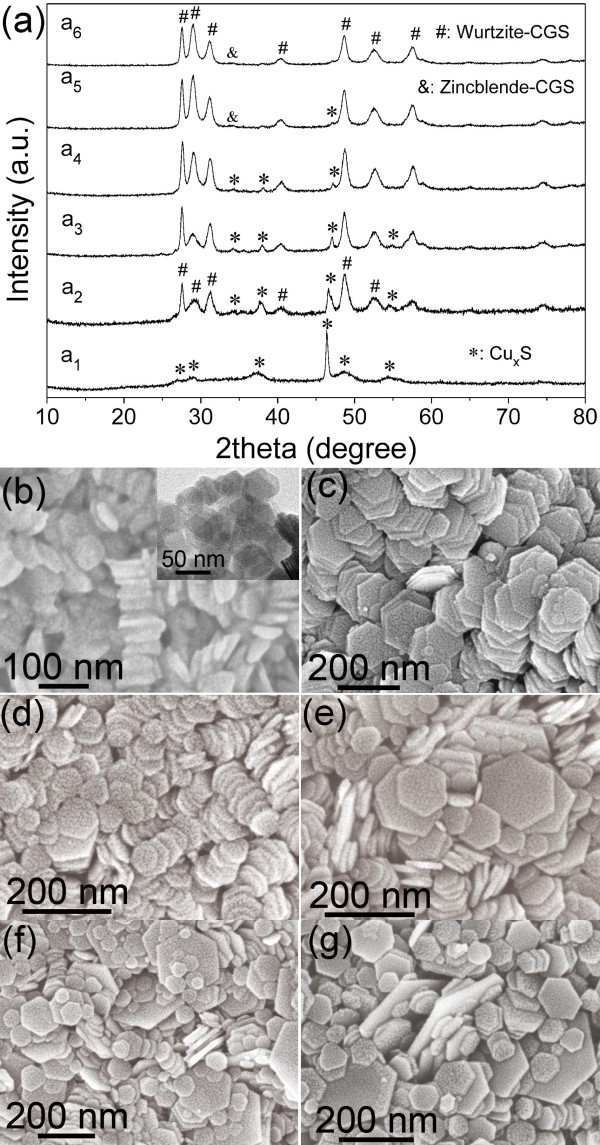
**XRD patterns (a) and SEM images (b, c, d, e, f, g) of samples collected at different reaction times.** (a_1_, b) 220°C, 0 min; (a_2_, c) 250°C, 0 min; (a_3_, d) 270°C, 0 min; (a_4_, e) 270°C, 10 min; (a_5_, f) 270°C, 20 min; (a_6_, g) 270°C, 30 min. The inset in b is the corresponding TEM image.

In our synthesis, metal chlorides (CuCl and GaCl_3_) could react with 1-dodecanethiol to form metal thiolates, which then decomposed into nanocrystals at elevated temperature [9,23]. When heating a mixture of CuCl, GaCl_3_, 1-dodecanethiol, and 1-octadecene to 140°C, a clear yellow solution formed, suggesting the formation of metal thiolates because of the reaction between metal chlorides and 1-dodecanethiol. Further heating the solution, the color of the solution gradually turned from clear yellow to dark gray (205°C), to dark, and finally to yellow. The color change implies nucleation and subsequent growth of nanocrystals due to the decomposition of as-formed metal thiolates. To investigate the growth process of CGS nanoplates, the samples collected at different reaction times were characterized by SEM, TEM and XRD, as shown in Figure 4. From Figure 4a (a_1_), it was surprisingly found that the sample collected at the early reaction stage was not CGS but binary copper sulfides (Additional file 1: Figure S2). As the reaction further proceeded, the samples mainly contain CGS along with the decrease of binary copper sulfides (Figure 4a (a_2_ to a_6_)). When the reaction was performed for 40 min, the product (Figure 1) was pure CGS nanoplates with a hardly detectable binary copper sulfide phase. Hence, in the growth process of CGS nanoplates, copper sulfides firstly formed, and then the as-formed copper sulfides were gradually phase-transformed to CGS nanoplates with proceeding of the reaction. The formation of copper sulfides in the early reaction stage maybe results from the difference of the reaction reactivity of two cationic precursors. From Figure 4b,c,d,e,f,g, it was clearly observed that all these intermediate samples were hexagonal nanoplates and the diameter of the nanoplates became uneven with the prolonged reaction, which may be due to the Ostwald ripening growth process.

**Figure 5:**
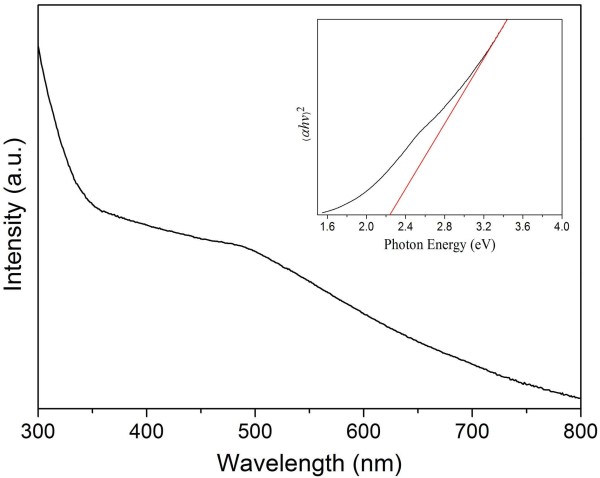
**Absorption spectrum of as-synthesized CuGaS**_**2 **_**nanoplates.** The bandgap is determined from the plot of (*αhv*)^2^*vs*. photon energy (shown in the inset).

Finally, the ultraviolet–visible absorption spectrum of as-synthesized CGS nanoplates has been measured at room temperature, as shown in Figure 5. A broad shoulder in the absorption spectrum can be observed at approximately 490 nm. According to the absorption spectrum, the optical bandgap of CGS can be estimated by using the equation of (*αhv*)^*n*^ = B(*hν* - *E*_g_), where *α* is the absorption coefficient, *hν* is the photo energy, B is a constant, *E*_g_ is optical bandgap, and *n* is either 1/2 for an indirect transition or 2 for a direct transition. As a direct bandgap semiconductor, the optical bandgap of CGS was estimated by extrapolating the linear region of a plot of (*αhv*)^2^ versus *hv* (shown in the inset of Figure 5). The estimated optical bandgap of as-synthesized CGS nanoplates is 2.24 eV. The bandgap is smaller than the literature value for wurtzite or zincblende CGS [20], which may be caused by the copper-rich composition of the as-synthesized nanoplates.

## Conclusions

In summary, we have developed a facile one-pot method to synthesize CuGaS_2_ nanoplates, wherein the mixed solution of CuCl, GaCl_3_, and *n*-dodecanethiol was thermally decomposed in non-coordinating solvent 1-octadecene at elevated temperature. The as-synthesized CuGaS_2_ nanoplates adopt a unique crystal structure of wurtzite-zincblende polytypism. In the growth process of CuGaS_2_ nanoplates, copper sulfides firstly formed, and then the as-formed copper sulfides were gradually phase-transformed to CGS nanoplates with proceeding of the reaction. The optical bandgap energy of the nanoplates is estimated to be approximately 2.24 eV. Our results will aid in the application of two-dimensional CuGaS_2_ nanoplates and the synthesis of other multicomponent sulfide nanomaterials.

## Competing interests

The authors declare that they have no competing interests.

## Authors’ contributions

ZPL planned and performed the experiments, collected and analyzed the data, and wrote the paper. KBT supervised the project, analyzed the results, and wrote the paper. QYH and LLW helped with the synthesis of the materials and the collection of the data. RT did the Rietveld fit of the obtained polytypic nanoplates. All the authors discussed the results and commented on the manuscript. All authors read and approved the final manuscript.

## Supplementary Material

Additional file 1 **Three crystal structure models of CuGaS2 and an XRD pattern of an intermediate sample. Figure S1.** Three crystal structure models of CuGaS_2_**(a)** tetragonal chalcopyrite structure; **(b)** cation-disordered cubic zincblende modification, **(c)** cation-disordered hexagonal wurtzite phase. **Figure S2.** XRD pattern of a sample collected at 220°C for 0 min. In the present case, Cu2-xS (JCPDS 23–0959) seems to contribute to the experimental pattern. Click here for file
