# Supplementary material for: Facile one-pot synthesis of polytypic CuGaS2 nanoplates
Source: Nanoscale Res Lett. 2013 Dec 13;8(1):524. doi: 10.1186/1556-276X-8-524 (PMC4029446; doi:10.1186/1556-276X-8-524)
Supplement: Additional file 1 — Three crystal structure models of CuGaS2 and an XRD pattern of an intermediate sample. Figure S1. Three crystal structure models of CuGaS2(a) tetragonal chalcopyrite structure; (b) cation-disordered cubic zincblende modification, (c) cation-disordered hexagonal wurtzite phase. Figure S2. XRD pattern of a sample collected at 220°C for 0 min. In the present case, Cu2-xS (JCPDS 23–0959) seems to contribute to the experimental pattern. [file 1556-276X-8-524-S1.doc]

# Supporting Information

# Facile one-pot synthesis of polytypic CuGaS_2_ nanoplates

*Zhongping Liu^1^, Qiaoyan Hao^1^, Rui Tang^2^, Linlin Wang^1^ and Kaibin Tang^1*^*

^1^Division of Nanomaterials and Chemistry, Hefei National Laboratory for Physical Sciences at the Microscale; Department of Chemistry, University of Science and Technology of China, Hefei 230026, P. R. China.

^2^Shanghai Institute of Applied Physics, Chinese Academy of Sciences, 2019 Jia Luo Road, Jiading District, Shanghai 201800, P. R. China

^*^Corresponding author: kbtang@ustc.edu.cn

**Figure S1** Three crystal structure models of CuGaS_2_: (a) tetragonal chalcopyrite structure; (b) cation-disordered cubic zincblende modification, (c) cation-disordered hexagonal wurtzite phase.

Crystal data

| Formula | CuGaS_2_ | |
| --- | --- | --- |
| Crystal system | Wurtzite | Zincblende |
| Space group | *P*6_3_mc (No. 186) | *F*-43m (No. 216) |
| Unit cell dimensions | *a* = *b* = 3.727(5) Å,  *c* = 6.197(6) Å | *a* = *b* = *c* = 5.309(0) Å |

Atomic coordinates

| **Atom** | **site** | **x/a** | **y/b** | **z/c** | **S.O.F.** |  | **Atom** | **site** | **x/a** | **y/b** | **z/c** | **S.O.F.** |
| --- | --- | --- | --- | --- | --- | --- | --- | --- | --- | --- | --- | --- |
| S | 2b | 1/3 | 2/3 | 0 | 1 |  | S | 4c | 1/4 | 1/4 | 1/4 | 1 |
| Ga | 2b | 1/3 | 2/3 | 3/8 | 0.5 |  | Ga | 4a | 0 | 0 | 0 | 0.5 |
| In | 2b | 1/3 | 2/3 | 3/8 | 0.5 |  | In | 4a | 0 | 0 | 0 | 0.5 |


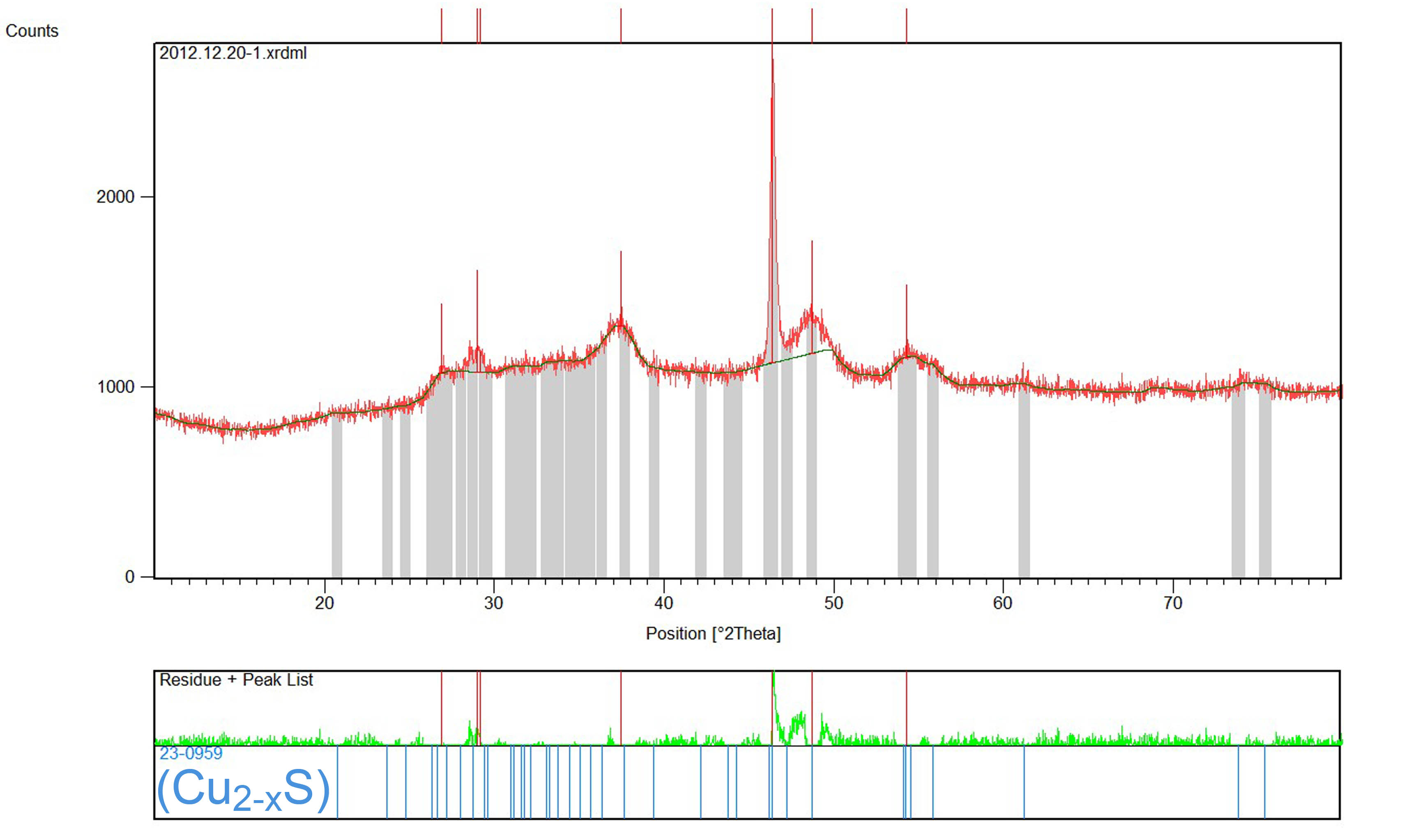


**Figure S2** XRD pattern of a sample collected at 220 ^o^C for 0 min. In the present case, Cu_2-x_S (JCPDS 23-0959) seems to contribute to the experimental pattern.
